# Investigating Turf Burn–Related Videos on TikTok: Cross-sectional Study

**DOI:** 10.2196/36218

**Published:** 2022-04-22

**Authors:** Brendan Jae Uk Hong, Benjamin K P Woo

**Affiliations:** 1 College of Osteopathic Medicine of the Pacific Western University of Health Sciences Pomona, CA United States; 2 Olive View-University of California Los Angeles Medical Center Sylmar, CA United States

**Keywords:** turf burn, skin, burn, turf, TikTok, misinformation, dermatologist, medical advice, peer support, companionship, web-based platform, sports medicine, dermatology, sports, sport, social media, mental health, sports injuries, athletic injuries, sport injury, athletic injury, athlete, injury, injuries, web-based video, psychiatry

## Abstract

**Background:**

Due to the increased use of artificial turf, turf burn has become a common sports injury. Turf burn is caused by exposed skin sliding on artificial turf. Health complications, such as methicillin-resistant *Staphylococcus aureus* outbreaks, sepsis, and pneumonia, have been linked to untreated turf burns, and many athletes have been turning to social media for advice and companionship regarding their sports injuries.

**Objective:**

The goal of this study is to categorize and quantitatively assess the percentage of turf burn–related posts on TikTok based on creator type, content, athletes’ experiences, and treatment and prevention methods. With these data, we not only investigate if there is room for health care professionals to assist in the distribution of evidence-based health education to athletes to counteract misinformation but also investigate if there is a potential audience of athletes on TikTok who have the potential to develop problematic responses to injuries.

**Methods:**

By using the *Discover* page on TikTok, we searched for the term *turf burn* on October 17, 2021. In total, 100 videos were analyzed. Videos were categorized and analyzed based on creator type, content, experiences of the athletes, and treatment and prevention methods. The number of likes and comments was recorded.

**Results:**

Most videos (98/100, 98%) were created by athletes. A small number of videos (2/100, 2%) were created by health care professionals. In terms of content, most videos (67/100, 67%) displayed turf burns. A small amount of videos (15/100, 15%) showed the incidents when turf burns were acquired, while around one-quarter of the videos (23/100, 23%) demonstrated the treatment and prevention of turf burns. Of the 23 treatment and prevention videos, a minority (4/23, 17%) showed the preferred treatment of turf burns, while most videos (19/23, 83%) showed nonpreferred treatments. The smallest amount of videos (2/100, 2%) were about turf burn education. Most of the videos created by athletes (56/98, 57%) depicted the negative experiences that patients had with turf burns. Some videos (37/98, 38%) depicted neutral experiences, while the smallest amount of videos (5/98, 5%) depicted positive experiences.

**Conclusions:**

Our study suggests that there is a potential audience of athletes on TikTok who could develop problematic responses to sports injuries, such as turf burns, as most of the people who post videos are athletes, and many of the posts demonstrate negative experiences associated with turf burns. TikTok is a growing social media platform that should be studied to determine if it can be used to create a social support group for injured athletes to prevent the progression of negative emotional responses into problematic responses. Physicians should also have a role in establishing their social media presence on TikTok and offering evidence-based advice to athletes while disproving misinformation on TikTok.

## Introduction

Due to increased artificial turf use, turf burn has become a common sports injury. Turf burn is caused by exposed skin sliding on artificial turf. Untreated turf burns are associated with methicillin-resistant *Staphylococcus aureus* (MRSA) outbreaks, sepsis, and pneumonia [1]. Many athletes turn to social media for advice regarding their injuries, since they experience emotions of sadness, irritation, anger, and frustration; isolation; a lack of motivation; sleep disturbances; and disengagement. These emotions and experiences can persist or worsen, leading to problematic responses for which athletes should seek help [2]. TikTok is a growing social media platform that is used to share personal experiences and education [3]. Our study aims to categorize and quantitatively assess the percentage of turf burn–related posts on TikTok based on creator type, content, athletes’ experiences, and treatment and prevention methods. We investigate if there is room for health care professionals to distribute evidence-based health education to athletes to counteract misinformation and if there is a potential audience of athletes on TikTok who may develop problematic responses due to their injuries.

## Methods

By using the *Discover* page on TikTok—a page designed for searching and exploring TikTok content by using keywords—we searched for the term *turf burn* and used the tab labeled *top* on October 17, 2021. This showed trending videos at that time. We used the first top 100 videos; each video was from a different user, and all videos were posted within March 2021 to October 2021. Videos were categorized and analyzed based on creator type, content, athletes’ experiences, and treatment and prevention methods. In addition, information in treatment and prevention videos was compared to clinical information by using UpToDate (UpToDate, Inc)—an evidence-based medical resource—and turf burn guidelines from the article “Athletic Skin Injuries” published by the peer-reviewed medical journal The Physician and Sportsmedicine [4,5]. One reviewer (BJUH) determined if the treatment and prevention video content was a preferred or nonpreferred form of treatment and prevention, while another reviewer (BKPW) independently reviewed the videos. The categorizations were all agreed upon by both evaluators, with no disagreements. Videos depicting a positive experience included videos showing injury improvement and treatment and prevention benefits. Videos depicting a negative experience included videos showing emotions that could develop into problematic responses [2]. Neutral videos consisted of videos in which athletes showed no overt opinions about their turf burns. The number of likes and comments was recorded.

## Results

Contentwise, most videos (67/100, 67%) displayed turf burns. A small amount of videos (15/100, 15%) showed the incidents when turf burns were acquired, while nearly one-quarter of the videos (23/100, 23%) demonstrated the treatment and prevention of turf burns. The smallest amount of videos (2/100, 2%) were about turf burn education (Table 1). Athletes created most of the videos (98/100, 98%), while health care professionals created a small fraction of the videos (2/100, 2%). Most athlete-created videos (56/98, 57%) depicted athletes’ negative experiences with turf burn. Some videos (37/98, 38%) depicted neutral experiences, while the smallest amount of videos (5/98, 5%) depicted positive experiences. Of the 23 treatment and prevention videos, a minority (4/23, 17%) showed the preferred treatment methods for turf burns, while most (19/23, 83%) showed nonpreferred treatments (Table 2). There was no mention of wound cleaning frequency in any video.

**Table 1:** Analysis of content in turf burn–related TikTok videos.

Video subject material	Videos (N=100), n	Comments (N=38,207), n (%)	Likes (N=2,428,435), n (%)
Displays turf burn	67	34,248 (89.6)	2,234,054 (92)
Incident of turf burn	15	724 (1.9)	85,550 (3.5)
Treatment and prevention	23	33,587 (87.9)	2,036,033 (83.8)
Education	2	129 (0.3)	14,452 (0.6)

**Table 2:** Analysis of turf burn–related videos on TikTok based on creator type, content, athletes’ experiences, treatment and prevention type, and methods for treatment and prevention.

Characteristic		Videos, n (%)	Comments, n (%)	Likes, n (%)
**Creator type**		100 (100)	38,207 (100)	2,428,435 (100)
	Athlete	98 (98)	38,078 (99.7)	2,413,983 (99.4)
	Health care professional	2 (2)	129 (0.3)	14,452 (0.6)
**Athletes’ experiences**		98 (100)	37,166 (100)	2,391,925 (100)
	Positive experience	5 (5.1)	102 (0.3)	8298 (0.4)
	Negative experience	56 (57.1)	35,901 (96.6)	2,287,253 (95.6)
	Neutral experience	37 (37.8)	1163 (3.1)	96,374 (4.0)
**Treatment and prevention type**		23 (100)	33,587 (100)	2,036,033 (100)
	Preferred treatment and prevention	4 (17.4)	148 (0.4)	20,816 (1)
	Nonpreferred treatment and prevention	19 (82.6)	33,439 (99.6)	2,015,217 (99)
**Cleaning method**		17 (100)	N/A^a^	N/A
	Washing with soap and water^b^	3 (17.6)	N/A	N/A
	Washing with water alone^b^	0 (0)	N/A	N/A
	Washing with normal saline^c^	0 (0)	N/A	N/A
	Washing with full-strength hydrogen peroxide^b^	14 (82.3)	N/A	N/A
	Washing with a diluted 50% hydrogen peroxide and 50% water solution^b^	0 (0)	N/A	N/A
	Washing with other cleaning solution	0 (0)	N/A	N/A
**Bandage method**		3 (100)	N/A	N/A
	Saline dressing^c^	1 (33.3)	N/A	N/A
	Petrolatum^c^	0 (0)	N/A	N/A
	Single antibiotic ointment^b^	0 (0)	N/A	N/A
	A combination of antibiotic ointments^c^	1 (33.3)	N/A	N/A
	Cotton pads^b^	1 (33.3)	N/A	N/A
**Prevention method**		2 (100)	N/A	N/A
	Protective clothing^c^	2 (100)	N/A	N/A

^a^N/A: not applicable.

^b^Nonpreferred treatment.

^c^Preferred treatment.

## Discussion

Our study found a disproportionate amount of nonpreferred methods for turf burn treatment and prevention. The majority of the treatment videos (19/23, 83%) demonstrated nonpreferred ways of treating and preventing turf burn, and these videos had the most comments (33,439/33,587, 99.6%) and likes (2,015,217/2,036,033, 99%). Most of these videos (14/23, 61%) showed the use of hydrogen peroxide to treat turf burns. Hydrogen peroxide is cytotoxic and can delay wound healing and increase the risk of complications [4]. MRSA-related turf burn injuries have been shown to be linked to hospitalized cases of cellulitis, septic arthritis, and abscesses, and proper wound care has been shown to decrease the risk of turf burn injury complications [1]. Consequently, physicians should establish a social media presence on TikTok and offer evidence-based treatment advice to athletes with turf burns, such as using a saline solution and dressings that provide a moist environment [4,5]. Furthermore, they should disprove misinformation to create awareness of common wound care mistakes. Future studies should examine the methods that health care professionals on TikTok use to interact with athletes and the methods used to refute misinformation. In addition, our study found that most people who post turf burn–related TikTok videos are athletes (98/100, 98%), and many posts (56/98, 57%) demonstrated negative emotional experiences associated with turf burns. The majority of videos (50/98, 51%) depicted an athlete feeling angry and frustrated due to pain from a turf burn. Pain can prevent athletes from participating in their sports and competing at their highest level, and negative emotions increase the likelihood of developing a problematic response to sports-related injuries, such as depression and substance abuse [2]. Because many of the videos depicted a negative experience resulting from a turf burn (56/98, 57%) and athletes created most of the videos (98/100, 98%), there is a potential group of athletes on TikTok who could develop problematic responses to their injuries, including turf burns, and could benefit from obtaining guidance from a physician. One limitation we encountered in this study was that we could not obtain information regarding the countries from which the TikTok videos were posted, as TikTok users do not have access to this information. As a result, more research is needed to investigate if there are any cultural differences in how athletes react to turf burn injuries and other sports injuries. There should also be future studies that investigate if physicians on TikTok and injured athlete support groups on TikTok can play a role in preventing the development of problematic responses to sports injuries.

## Abbreviations

MRSA: methicillin-resistant *Staphylococcus aureus*
